# Use of MRI in the diagnosis and prognosis of acute necrotizing encephalopathy in a Chinese teenager: A case report

**DOI:** 10.1097/MD.0000000000017797

**Published:** 2019-11-01

**Authors:** Hua Li, Caihong Sun, Shaohua Chi, Yan Wang, Lin Wu, Xia Qin

**Affiliations:** a Department of Magnetic Resonance Imaging; b Department of Intensive Care Unit; c Department of Neurology, People's Hospital of RiZhao, Rizhao, China.

**Keywords:** acute necrotizing encephalopathy, corticosteroid, methylprednisolone, oseltamivir

## Abstract

**Rationale:**

Acute necrotizing encephalopathy (ANE) is a rapidly progressing disease associated with frequent neurologic sequelae and has poor prognosis. Currently, the diagnosis and treatment of ANE rely on neuroradiologic findings and offering supportive care. Here, we report the successful treatment of a teenager diagnosed with ANE using combination of high-dose methylprednisolone and oseltamivir.

**Patient concerns:**

The patient, a 15-year-old female, presented with impaired consciousness and seizures secondary to acute upper respiratory tract infection. A series of brain magnetic resonance images (MRIs) were obtained toward establishing a possible diagnosis.

**Diagnosis:**

Based on the history of presenting illness and subsequent brain MRI scans, the patient was diagnosed to be suffering from ANE.

**Interventions:**

Following the diagnosis, the patient was placed on therapy comprising of high-dose methylprednisolone and oseltamivir.

**Outcomes:**

After treatment with methylprednisolone and oseltamivir for 15 days, the patient recovered nearly completely from ANE as confirmed by subsequent brain MRI scans. No complications or other emerging clinical symptoms were noted for the duration of follow-up that lasted 6 months.

**Lessons:**

Contrary to common reports, ANE can occur beyond pediatric populations and its treatment should not be restricted to supportive care. Our case suggests that the use of high-dose corticosteroids and oseltamivir leads to promising prognosis.

## 1 Introduction

Acute necrotizing encephalopathy (ANE) is a medical condition characterized by multifocal symmetric lesions involving the thalami, brain stem, cerebellum, and white matter.^[1]^ It has been widely known that ANE mainly occurs in children with clinical symptoms often resembling those of upper respiratory tract infections^[2]^ and influenza encephalopathy such as sudden onset of high fever, severe convulsion, and dramatic neurologic deficits that rapidly progress to coma.^[3]^ Although mostly described in Japanese population,^[4]^ emerging studies indicate that ANE is not limited to a particular race.^[5–7]^ The precise pathogenesis of ANE remains unclear and there is no specific treatment.^[8]^ In this case report, we identified the typical features of ANE using magnetic resonance image (MRI) in a female teenager and applied treatment strategy involving high-dose corticosteroids and oseltamivir.

## 2 Ethics approval and patient consent

The study was approved by the ethical committee of People's Hospital of RiZhao (approval number: 2019006), and signed informed consent to publish this case report was granted by the parents to the patient.

## 3 Case report

### 3.1 History

The patient is a 15-year-old female whose medical history did not reveal any previous incidence of severe illness. Six days prior to admission, the patient had experienced high fever (highest temperature of 39.5°C) and mild signs of upper respiratory infection. At the time of admission, she was experiencing episodes of unconsciousness.

### 3.2 Presentation

The patient manifested altered mental status and seizures. She was unable to response to sound and had lost control of her eye reflexes. Moreover, the patient's limbs twitched accompanied with trismus and she experienced nausea and vomiting, but not urinary incontinence. The earlier mentioned symptoms lasted for 1 to 3 minutes and disappeared spontaneously and reoccurred in episodes. A computed tomography scan, which showed symmetrical brain lesions in the cerebellum and thalami, was performed on the patient at the local hospital before being admitted to the department of our hospital.

### 3.3 Examination

The blood pressure of the patient was 115/75 mm Hg and she displayed a positive left Kerning sign. Other physical examination results were unremarkable. Hematologic data for the patient are presented in Table 1. Analysis of the cerebrospinal fluid (CSF) following lumbar puncture found a clear, colorless fluid with an opening pressure of 310 cmH_2_O. Other results are summarized in Table 2.

**Table 1:**
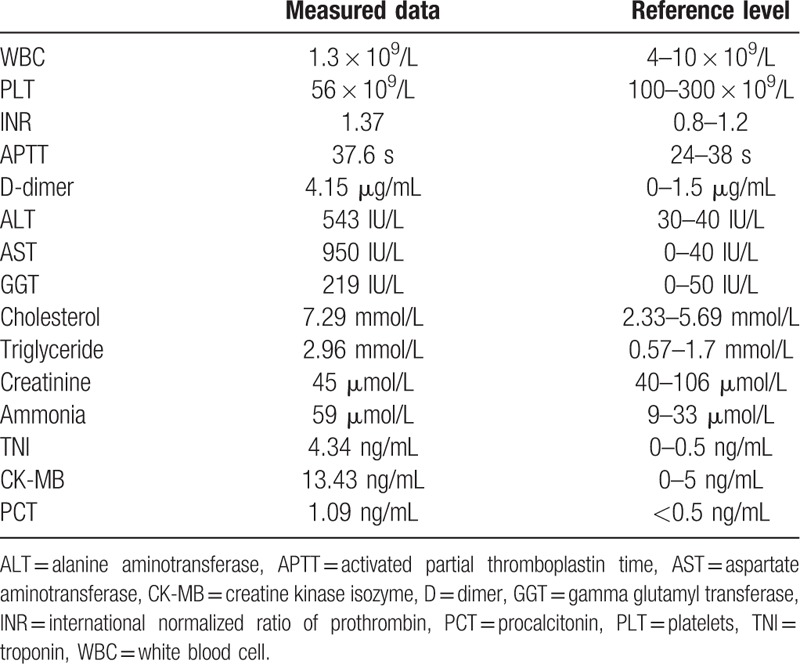
Hematologic and blood biochemistry data.

**Table 2:**
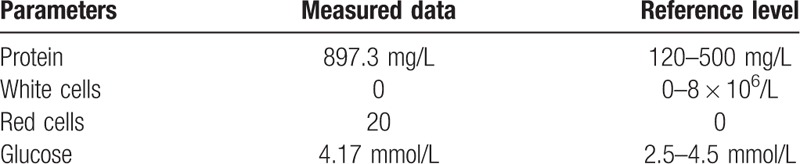
Analysis of cerebrospinal fluid.

**Figure 1:**
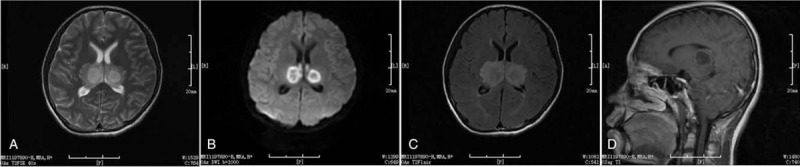
Brain magnetic resonance imaging of a 15-year-old female. The scans showed multiple symmetrical brain lesions (slightly ring enhancing) in bilateral dorsal areas of the thalami, dorsal caps of the pontine, and bilateral cerebellar hemispheres. (A) T2. (B) Diffusion-weighted imaging. (C) T2 flair. (D) T1 enhanced.

Brain MRI revealed multiple symmetrical brain lesions. Prolonged T1- and T2-relaxation time signals were evident in the right regions of the frontal and parietal lobes, bilateral dorsal areas of the thalami, dorsal caps of the pontine, bilateral cerebellar hemispheres, and the cauda cerebelli. High signal intensities and slightly low central signals were demonstrated on diffusion-weighted imaging (DWI). After enhancement, the lesions of the bilateral dorsal thalamus, bilateral cerebellar hemisphere, and right parietal lobe exhibited low-level circular enhancement (Fig. 1).

### 3.4 Diagnosis and treatment

**Figure 2:**
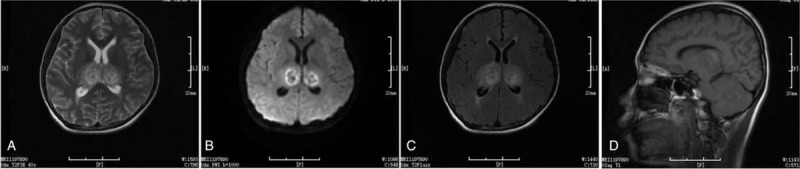
Magnetic resonance imaging of the same patient 7 days after diagnosis with acute necrotizing encephalopathy. The scans showed that lesions of the bilateral dorsal thalamus are slightly reduced, the internal signal obviously uneven, and having short T1 signals. (A) T2. (B) Diffusion-weighted imaging. (C) T2 flair. (D) T1.

**Figure 3:**
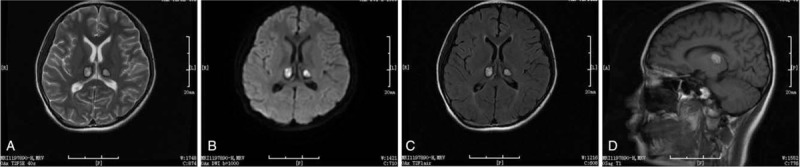
Magnetic resonance imaging of the same patient 15 days after diagnosis with acute necrotizing encephalopathy. The scans showed reduction in the lesions of bilateral dorsal area of the thalami, dorsal caps of the pontine, and bilateral cerebellar hemispheres. Short T2 signals around the lesions are also evident. (A) T2. (B) Diffusion-weighted imaging. (C) T2 flair. (D) T1.

Based on the characteristic MRI findings, clinical presentations, the history of upper respiratory tract infection, and the lack of other prominent etiologies, a diagnosis of virus-associated ANE was made. In addition to maintaining sufficient hydration, oseltamivir (150 mg twice daily for 7 days), methylprednisolone (450 mg once daily for 5 days), and acyclovir (500 mg thrice daily for 10 days) were administered to the patient intravenously. Magnesium isoglycyrrhizinate, mannitol, and other supporting treatments were also given to the patient as needed. On the 7th day of hospitalization, the patient regained consciousness and was responsive to various cues. MRI scans at this timepoint revealed that the size of lesions in the bilateral dorsal thalamus were slightly reduced, the internal signals were obviously uneven and surrounded by short T1 signals (Fig. 2). The dose of oseltamivir was then reduced to 75 mg for a further 7 days, and methylprednisolone was gradually reduced to 80 mg daily. Prednisone acetate (1.5 mg/kg/d for 3 months, dose reduced week by week) was then given to the patient in decreasing doses until completely withdrawal of the medication. This treatment strategy led to a near complete recovery from ANE as confirmed by MRI findings 15 days after diagnosis of the disease (Fig. 3). The size of lesions in the bilateral dorsal regions of the thalami, dorsal caps of pontine, and bilateral cerebellar hemispheres were significantly reduced and surrounded by short T2 signals.

### 3.5 Equipment and procedures

The GE Signa Excite 1.5T superconducting MRI system and orthogonal coils were used in this study. The patient underwent both plain and enhanced MRI scanning. The scanning parameters employed include a layer thickness of 6 mm, interval of 2 mm, and field of view of 18.0 × 18.0 cm. Others are horizontal spin-echo T1-weighted imaging (SET1WI) (TR: repetition time 300–500 milliseconds, TE: echo time 8–12 milliseconds), fast spin echo T2-weighted imaging (FSET2WI) (TR: 2500–5000 milliseconds, TE: 90–102 milliseconds), fluid-attenuated inversion recovery (FLAIR) (TR: 8002 milliseconds, TE: 104 milliseconds) and diffusion-weighted echo-planar imaging (TR: 7100 milliseconds, TE: 129 milliseconds). Additional parameters include sagittal SET1WI (TR: 300–500 milliseconds, TE: 8–12 milliseconds), and partial coronal scanning FSET2WI (TR: 2500–5000 milliseconds, TE: 90–102 milliseconds). For the enhanced MRI scanning, the contrast agent gadolinium-diethylenetriamine pentaacetic acid 0.1 mL/kg was administered intravenously. The transverse, sagittal, and coronal positions were SETWI (TR: 300–500 milliseconds, TE: 8–12 milliseconds).

## 4 Discussion

The ANE is a rare central nervous system complication associated with influenza or other viral infections and is accompanied by high morbidity. The condition is mainly reported in pediatric populations in Japan, the 1st case having been described by Mizuguchi and co-workers in 1995.^[4]^ Currently, over 70 cases of ANE have been reported around the world. In the present case report, ANE involve a patient who is much older than the average age of cases that have been previously reported.^[4,9,10]^

The clinical presentation of ANE is heterogeneous. In many cases, especially severe ones, patients often developed sudden deterioration in mental status after presenting with severe symptoms of virus infection. Organ dysfunction in the form of acute lung injury, hepatic failure, and/or shock,^[11]^ and widespread brain edema among these patients usually lead to poor prognosis.^[12–14]^ Elevated opening pressure can be seen during lumbar puncture, but CSF analysis usually reveals normal cell counts and glucose levels with occasional mild elevation of protein levels.^[6,15]^ In other cases, systemic manifestations are less pronounced. In this case report, the patient manifested classic signs of preceding infection, followed by progressive central nervous system deficits. Other signs and symptoms consistent with earlier reports include liver dysfunction, myocardial damage, thrombocytopenia, and leucopenia.

Despite increasing studies and reports on ANE in recent years, the pathogenesis of the disease remains unclear. It is generally hypothesized that the “cytokine storm” in response to the viral infection induces systemic inflammation that may cause the observed encephalopathy.^[13]^ The ensuing hypercytokinemia thought to elicit various immune responses, which cause endothelial cell damage, bringing about changes in the permeability of the vascular endothelium and structural disruption to the blood–brain barrier.^[16]^

Typical MRI features in the diagnosis of ANE include multiple focal lesions of edematous necrosis that are symmetrically distributed in the bilateral thalami. As such, the area will exhibit high T1WI signals. Other brain regions such as the putamina, cerebral and cerebellar deep white matter, and brainstem tegmentum are often also involved.^[17]^ If brainstem is involved, MR images will reveal high signals on T1WI and symptoms will be accompanied by hemorrhage.^[17]^ In other lesions, T1WI usually shows low signal intensity with T2WI and T2Flair displaying mixed high signals. The DWI usually shows a high signal, revealing multifocal, symmetrical involvement. After enhancement there is a slight circular enhancement and features of necrosis can be seen as the disease progresses.^[17,18]^

A study by Takeshita demonstrated that lesions can be observed using enhanced MRI even before neurologic symptoms of ANE appear.^[10]^ These lesions were, notably, unrecognized by normal MRI, thereby affirming the value of enhanced MRI in the early diagnosis of ANE. From this study, it was confirmed that the destruction of the blood–brain barrier and changes in vascular permeability were responsible for the initial progression of ANE. Subsequent plasma exudation, neural, gliacyte necrosis, and bleeding resulted in multiorgan dysfunction.

As mentioned earlier, ANE has a high morbidity and mortality.^[3,19]^ Neurologic sequalae are frequently observed in survivors.^[8]^ The prognosis of ANE can be predicted by serial MRI findings. For instance, observation of hemorrhage and local tissue loss on MRI predicts a poor prognosis.^[18]^ In our experience, we found that MRI also plays an important role in evaluating treatment efficiency. The lesions associated with ANE were observed to shrink during the treatment. ANE is often misdiagnosed as acute encephalopathy because of similarities in the clinical presentations between these 2 conditions. Unlike in ANE, the MRI of acute encephalopathy presents as cytotoxic edema, which produces high signal intensity on DWI.^[5,20]^ Due to the difficulty in obtaining biopsies, the diagnosis of ANE mainly relies on characteristic neuroradiologic findings.^[8]^ The MRI findings in the present case were consistent with those found in previously reported cases. In addition, the changes in MRI scans in our case also correlated with the clinical course of ANE.

Hiroyuki Yamamoto has developed the ANE severity score to predict the prognosis of ANE according to clinical variables that correlate with neurologic outcomes.^[21]^ The study suggested that poor outcomes are expected among patients suffering from coma or brainstem lesions, as well as those aged over 48 months. On the contrary, elevated CSF protein, low platelet counts, and liver dysfunction were not predictors of ANE prognosis.

Currently, there are no specific standardized treatment guidelines in the management of ANE. The general trend is to offer intensive care, and symptomatic and empirical treatments. The most commonly reported interventions are intravenous corticosteroids, antiviral treatment (e.g., oseltamivir), and gamma globulins.^[5,13]^ In the present case report, the patient received corticosteroids and antiviral treatment resulting in good clinical outcomes. Our approach confirms the potential to manage this rare but fatal condition and can be explored by other physicians.

## Author contributions

**Data curation:** ShaoHua Chi, Lin Wu, Xia Qin.

**Resources:** Lin Wu.

**Writing – original draft:** Hua Li, Caihong Sun.

**Writing – review & editing:** Yan Wang.
